# Structural basis of Ac-SDKP hydrolysis by Angiotensin-I converting enzyme

**DOI:** 10.1038/srep13742

**Published:** 2015-09-25

**Authors:** Geoffrey Masuyer, Ross G. Douglas, Edward D. Sturrock, K. Ravi Acharya

**Affiliations:** 1 Department of Biology and Biochemistry, University of Bath, Claverton Down, Bath BA2 7AY, UK; 2 Department of Integrative Biomedical Sciences and Institute of Infectious Disease and Molecular Medicine, University of Cape Town, Observatory 7935, South Africa

## Abstract

Angiotensin-I converting enzyme (ACE) is a zinc dipeptidylcarboxypeptidase with two active domains and plays a key role in the regulation of blood pressure and electrolyte homeostasis, making it the principal target in the treatment of cardiovascular disease. More recently, the tetrapetide *N*-acetyl-Ser–Asp–Lys–Pro (Ac-SDKP) has emerged as a potent antifibrotic agent and negative regulator of haematopoietic stem cell differentiation which is processed exclusively by ACE. Here we provide a detailed biochemical and structural basis for the domain preference of Ac-SDKP. The high resolution crystal structures of N-domain ACE in complex with the dipeptide products of Ac-SDKP cleavage were obtained and offered a template to model the mechanism of substrate recognition of the enzyme. A comprehensive kinetic study of Ac-SDKP and domain co-operation was performed and indicated domain interactions affecting processing of the tetrapeptide substrate. Our results further illustrate the molecular basis for N-domain selectivity and should help design novel ACE inhibitors and Ac-SDKP analogues that could be used in the treatment of fibrosis disorders.

Angiotensin-1-converting enzyme (ACE, EC 3.4.15.1) is a zinc peptidase and a key enzyme in cardio-vascular physiology^1^,^2^. ACE is a central component of the Renin-Angiotensin-Aldosterone System (RAAS), wherein it converts angiotensin I into the vasoactive peptide hormone angiotensin II (AngII)^3^,^4^. Interestingly, ACE consists of two catalytically active domains^5^,^6^ (referred to as the N- and C-domains based on location on the polypeptide chain) that, while very similar in sequence and structural topology, display differences in substrate processing abilities^6^,^7^,^8^. ACE is also able to cleave the vasodilatory peptide bradykinin and therefore enhance the blood pressure response^9^. In addition to the cleavage of vasopeptides, ACE is able to cleave a variety of peptides that are outside of its blood pressure role. While these are not the most well-known substrates for ACE, many of these have important physiological roles and therefore warrant careful study.

*N*-acetyl-Ser-Asp-Lys-Pro (Ac-SDKP) is specifically cleared by ACE^10^. This process has been reported to be carried out predominantly by the N-domain^7^. Ac-SDKP was originally discovered as a potent inhibitor of bone marrow derived stem cell differentiation through inhibition of the G1-S phase transition^11^,^12^. This suggested a possible role of Ac-SDKP as a co-treatment to reduce the treatment associated severity of some chemotherapies due to this peptide being able to keep stem cells in the quiescent state^13^. Later findings indicated that Ac-SDKP also possesses potent anti-fibrotic and anti-inflammatory effects in numerous tissues including heart, liver, kidney and lung^14^,^15^,^16^,^17^,^18^,^19^,^20^,^21^,^22^. Ac-SDKP is able to blunt the Smad signalling effects of transforming growth factor-β, a prominent fibrosis marker^23^,^24^. Thus, Ac-SDKP appears to have a prominent physiological role.

The N- and C-domains of ACE have been shown to display negative co-operativity in substrate hydrolysis^25^,^26^,^27^. While this is observed with many synthetic and naturally occurring peptides, not all substrates displayed such an effect^27^. To date, no studies have been performed on the physiologically relevant substrate Ac-SDKP.

The X-ray crystal structures of both individual homologous ACE domains have been previously determined^28^,^29^ and more recently the structure of N-domain in complex with the phosphinic tripeptide RXP407^30^ highlighted the structural requirements for domain specific inhibition. Furthermore, the structure of the C-domain in complex with AngII^31^ gave the first insight into the mechanism of peptide recognition by ACE and revealed the regulatory role of AngII on the enzyme’s activity. Studying the mechanisms of peptide recognition for each domain of ACE should therefore help our understanding of substrate selectivity and lead to the design of better inhibitors.

The purpose of this study was to understand the kinetic nature of Ac-SDKP hydrolysis and the structural basis for its interaction with the N-domain of ACE. The high resolution (1.8 Å) X-ray crystal structures of the N-domain in complex with the two products of Ac-SDKP cleavage, the dipeptides Ac-SD and KP, were determined. The extent of domain interaction during substrate hydrolysis and the domain selectivity of these processes were further assessed using a fluorescamine assay. Together the data reported here allowed the construction of a comprehensive model of N-domain selective substrate recognition and peptidase activity.

## Results

### Structure of the Ac-SDKP:N-domain complexes

**Figure 1:**
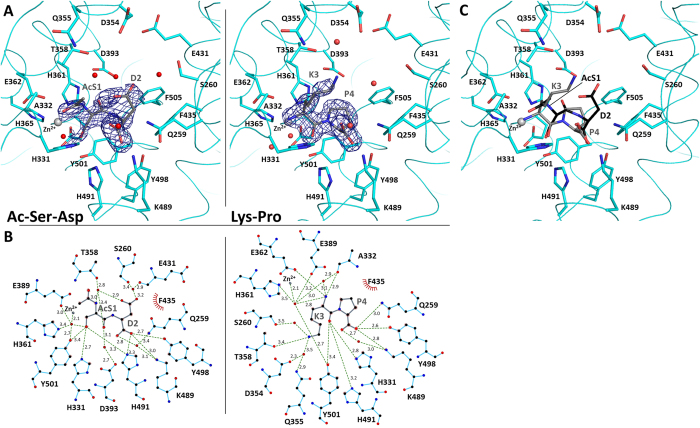
Mechanism of Ac-SDKP fragments binding to N-domain ACE. (**A**) Portions of the electron density map at the site of the bound peptides with left, Ac-SD, and right, KP. The maps were generated using REFMAC5^61^ and correspond to the difference weighted *2mFo-DFc* density map, contoured at 1.0σ level, in which the peptide atoms were omitted. N-dom in cyan (interacting residues in stick representation), bound dipeptides in grey stick, catalytic zinc ion and water molecules as grey and red spheres respectively. (**B**) Schematic of peptide binding to N-ACE. Interactions were calculated with LigPlot^+^^64^. (**C**) Superposition of the dipeptide Ac-SD and KP structures. Ac-SD and KP are presented in black and grey sticks, respectively.

Co-crystallisation experiments were carried out to assess the interaction between the N-domain and tetrapeptide Ac-SDKP. Surprisingly, two crystal forms were obtained (Table 1). One form presented the same cell dimensions as the previously described minimally glycosylated N-domain^30^ with 2 chains per asymmetric unit in *P1*. The structure at 1.8 Å showed electron difference density within the catalytic channel (Fig. 1A), which was interpreted as the Ac-SD product, resulting from the enzymatic reaction. The structure from the second crystal form was solved at a similar resolution, in space group *P1* with larger cell dimensions, fitting 4 molecules per asymmetric unit. No significant conformational changes were observed for the N-domain, with only the expected N-terminal hinge region showing signs of disorder in one of the four molecules. However, this crystal form did present an alternative and unambiguous difference electron density at the active site, with the map clearly showing the dipeptidyl carboxypeptidase product of Ac-SDKP hydrolysis (KP, Fig. 1A). The two different fragments occupy a similar position within the S1′-S2′ sub-pockets while only interacting with the zinc ion through water-mediated interactions.

The two peptides have their C-terminal end strongly anchored by hydrogen bonds with residues Lys489, Tyr498 and Gln259 forming the S2′ site, amino acids previously shown to be key anchoring residues (Fig. 1B). The aromatic Phe435 and Phe505 also stabilise this end of the peptide by hydrophobic interaction with the Pro or Asp side chains of the substrate. The acidic group of the substrate Asp showed signs of flexibility (Fig. 1A), but may make further water-mediated contacts with the backbone of surrounding enzyme residues Glu431, Thr358 and the Ser260 hydroxyl group. The main difference between the two peptidic substrate fragments resides at their first residue due to the presence of the *N*-acetyl group on the serine. With the Lys-Pro fragment, the lysine backbone follow a similar bonding pattern to what was observed in the C-domain:Ang II structure, with its oxygen being stabilised by multiple hydrogen bonds with enzyme residues His331, His491 and Tyr501 (Fig. 1B) and its amino group interacting with the catalytic site. The long side chain can make further polar connection directly with Thr358 and water bridges with Asp354, Gln355 and Ser260. On the other hand, the *N*-acetyl-serine (AcS), of the Ac-Ser-Asp fragment, presents a different orientation. Its Hydroxyl side chain makes direct electrostatic contacts with His331, His491 and Tyr501, as well as a water-mediated interaction with Zn^2+^. The AcS amino group interacts with a single water molecule itself connected to Thr358 (main chain) and Asp393. The acetyl moiety may be stabilised in the small hydrophobic area composed of Ala332 and Thr358.

### Kinetics of Ac-SDKP hydrolysis by ACE

**Figure 2:**
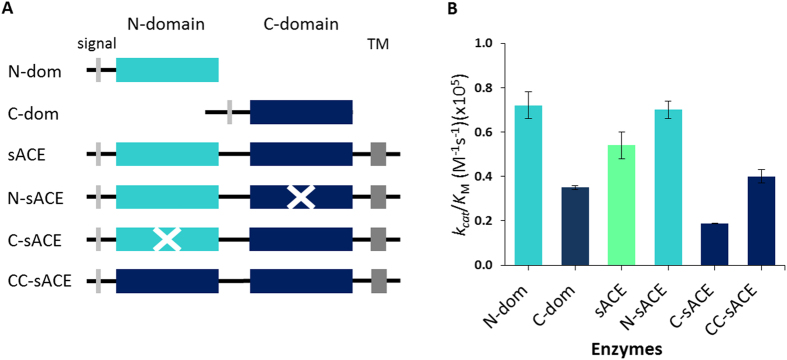
Analysis of ACE catalytic efficiency on Ac-SDKP. (**A**) Details of ACE constructs. The wild-type ACE (sACE) contains a signal peptide (light grey), the two homologous ectodomains (N-domain in cyan and C-domain in blue), the transmembrane domain (dark grey) is also indicated. Inactive domains are crossed over. (**B**) Catalytic efficiency of ACE on Ac-SDKP.

Kinetic analysis was performed to assess the selectivity and possible co-operativity of the N- and C-domains in Ac-SDKP processing. Individual N-domain and C-domain constructs (Fig. 2A) displayed comparable *K*_m_ values of 199.6 μM and 138.2 μM, respectively (Table 2). The individual N-domain had a turnover rate that was approximately 3-fold higher than that of the C-domain, confirming the N-domain preference for Ac-SDKP, albeit lower than original reports.

Wild-type sACE, with both domains active, displayed a *K*_m_ value of 239.5 μM while retaining a *k*_cat_ value (per mole active site) similar to the individual N-domain. Thus, the combined domain turnover rate of sACE (25.2 s^−1^) was slightly higher than the sum of the *k*_cat_ values for the individual domains.

In order to further delineate domain selectivity and assess the activity of domain active sites in full length sACE, C-sACE and N-sACE enzymes (these constructs are full length sACE molecules where one of the two active sites has been inactivated by mutation) were employed (Fig. 2A). Interestingly, the *K*_m_ values of the two domain (*X*-sACE) enzymes were approximately 3-fold higher than their single domain counterparts, and twice that of wild-type sACE, again showing an effect of domain interaction on substrate on-off rates. The *k*_cat_ values were approximately 2-fold higher in C-sACE and N-sACE constructs than their respective single domains, which may be indicative of an inter-domain influence on substrate release. Overall, this resulted in a similar catalytic efficiency between the individual N-domain and the same domain in full length form (N-sACE), whereas C-sACE was approximately half that of its single domain counterpart (that is, the individual C-domain, Fig. 2B).

So as to better appreciate the role of the C-domain in the full length molecule, the CC-sACE molecule, comprising two active C-domains connected with the interdomain linker (Fig. 2A), was characterised in terms of Ac-SDKP kinetics. This enzyme showed an increased *K*_m_ compared to wild-type sACE but in a similar range to that of the other full length constructs (Table 2). Interestingly, the turnover rate per unit active site of CC-sACE (19.4 s^−1^) showed a two-fold increase compared to C-sACE (8.2 s^−1^). The resulting improved catalytic efficiency therefore suggests a role of the N-domain in lowering C-domain activity in the full length somatic enzyme towards the substrate.

### Modelling of the Ac-SDKP substrate interaction with ACE

**Figure 3:**
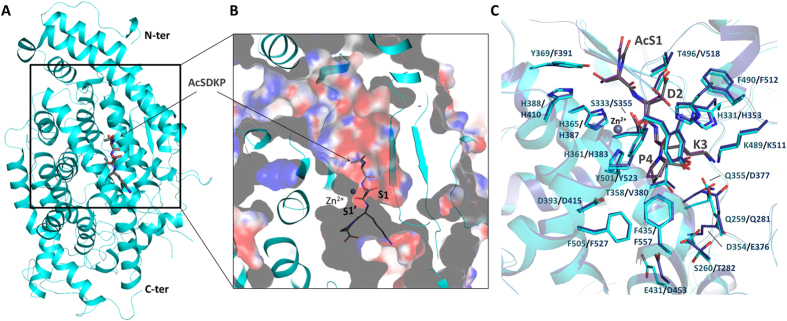
Modelling of Ac-SDKP binding to ACE. (**A**) Ac-SDKP was manually docked within the catalytic channel of N-dom based on the KP complex structure. The model was refined using the Rosetta FlexPepDock server^32^. N-dom is shown in cyan, and Ac-SDKP in grey stick. (**B**) Close up view of Ac-SDKP within the catalytic channel, surface charged was calculated with the PyMol/APBS tool^65^. (**C**) Comparison of Ac-SDKP models bound to N- and C-domain ACE. N-dom in cyan, C-dom in blue; Ac-SDKP in grey and purple in for the N- and C-domain models, respectively. Residues involved in substrate interaction are labelled with their protein respective colour.

**Figure 4:**
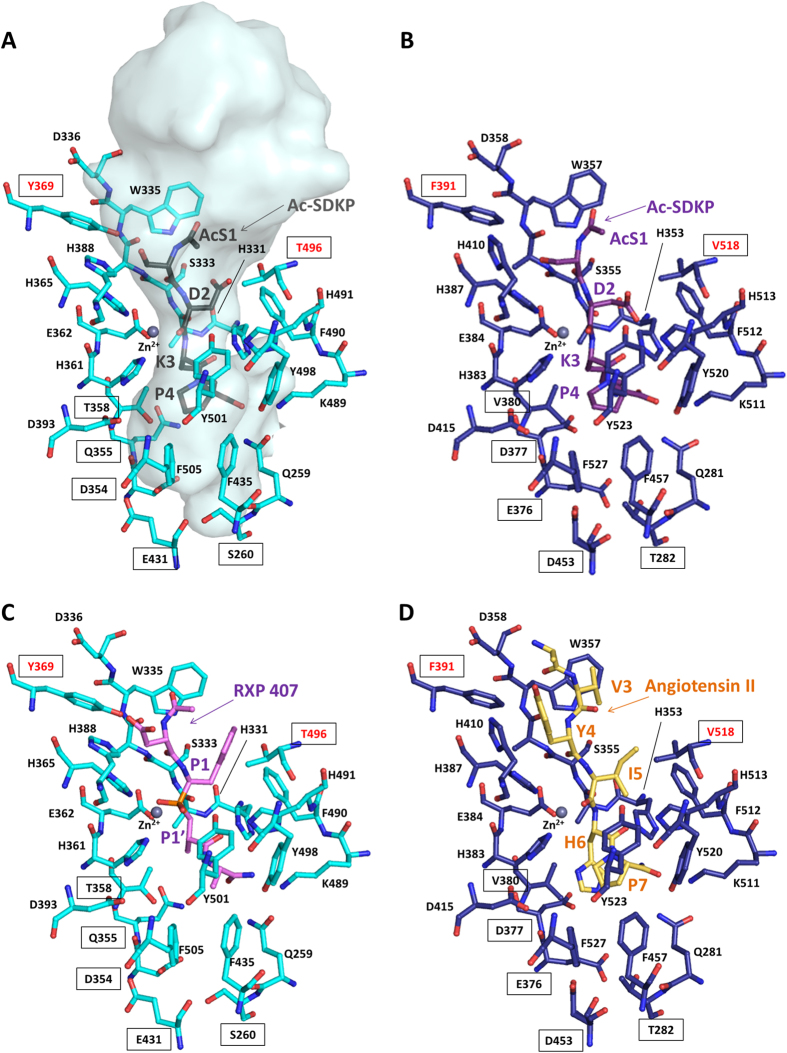
Comparison of domain specific substrate and inhibitors in ACE. (**A**) Model of Ac-SDKP binding to N-domain ACE (as above). The catalytic channel is represented with a transparent surface calculated with the program HOLLOW^66^ (**B**) Model of Ac-SDKP binding to C-domain ACE, with C-domain ACE in blue, and Ac-SDKP in purple. (**C**) Crystal structure of the N-domain specific RXP 407 inhibitor bound to N-domain ACE (PDB 3NXQ)^30^, with RXP 407 in pink. (**D**) Crystal structure of C-domain ACE in complex with AngII (PDB 4APH)^31^; with AngII in yellow. Non-conserved residues of the catalytic channel are indicated by a framed label. The residues involved in N-domain specificity^30^ are labelled in red.

The crystal structure of the N-domain with the KP fragment gave useful insight into the peptide binding mechanism and allowed us to propose a model for the full substrate interaction (Fig. 3). The lysyl-proline-bound structure was used as the basis of the superposition with the C-domain:AngII (inhibitory peptide resulting from angiotensin I cleavage) structure. The C-terminal end of the Ac-SDKP peptide presents a conserved peptide binding mechanism between the two domains and was thus the best anchor on which to build the complete model (Fig. 4). The C-domain:AngII structure also shows many of the same residues between the two domain interacting along the peptide backbone, so that the rest of the Ac-SDKP peptide was docked based on this mechanism. The Ac-SDKP models for both domains were then refined using the Rosetta FlexPepDock server (which uses a Monte-Carlo with minimization approach)^32^. The tetrapeptide fits well within the narrow catalytic channel by interacting with ACE domain active sites through its backbone from P2′ to P1, and additional contacts with the P2 Ser and the corresponding N-domain subsite. The acetyl group can be easily accommodated within the wide S2 pocket (Figs 3 and 4). In order to assess the preference of Ac-SDKP for the N-domain, the models were compared to the crystal structures of the N-domain with RXP 407 (an N-domain specific inhibitor^33^) and C-domain with AngII (which shows C-domain selectivity^34^) (Fig. 4). Previous structural analysis^30^ has highlighted the high degree of conservation between the two domains within the S’ binding sites (Table 3). This is important since it highlights the conservation of essential residues for a general role in substrate binding. The specificity for Ac-SDKP appears to reside mostly at the S2 subsite. This subsite, while cavernous, provides contacts in the N-domain that could explain partial N-domain preference of this substrate. There, the hydroxyl group of Ac-Ser is within hydrogen bonding distance of conserved residue His388. The non-conserved Tyr369 can also provide further electrostatic potential compared to the C-domain Phe391, and has been identified previously as a key residue for ligand selectivity^35^,^36^. Furthermore, the polar (N-domain) Thr496 may provide additional interaction with the substrate Asp side chains compared to the C-domain enzyme residue Val518. In the P1′ pocket, our structure with Lys-Pro shows that the dipeptide’s ε-amino group can interact with Thr358 which is also not conserved in C-domain ACE (Val380). It should be added that water molecules are expected to play a significant role in peptide binding, as illustrated by several water-mediated bonds in the crystal structures presented here (Fig. 1), but were not included in the models for practical reasons.

**Figure 5:**
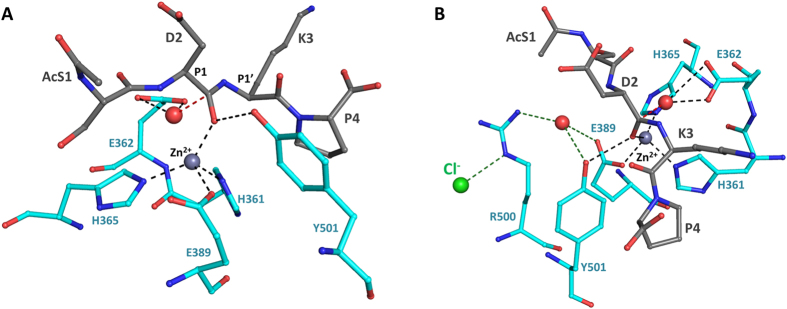
Proposed model for the catalytic mechanism of Ac-SDKP hydrolysis by N-domain ACE. (**A**) Model of Ac-SDKP (grey) bound to N-domain ACE (cyan) prepared as above. N-domain ACE residues involved in the zinc-mediated activity and stabilisation of the potential transition state intermediates are shown as stick. The water involved in the nucleophilic reaction is shown in red (sphere). (**B**) Potential role of the chloride ion in activity. Chloride ion is shown in green, with potential hydrogen bond and water mediated-interactions with the catalytic site highlighted as green dashes.

ACE belongs to the gluzincin family of metalloproteases and catalysis is expected to follow a general base-type mechanism. The structures determined here allow us to more accurately predict the substrate hydrolysis mechanism employed by ACE. The cleavage of Ac-SDKP is predicted to happen after displacement of the zinc-bound water molecule which then attacks the scissile carbonyl bond to form an oxyanion (Fig. 5). The nucleophilic attack is enhanced with the water being coordinated by the active site Glu362. The resulting intermediate is likely stabilised by Tyr501. As mentioned previously, chloride has an important effect on ACE activity which is also substrate-dependent in the C-domain^37^. The function of chloride in N-ACE is less understood, but it is unlikely to affect substrate specificity. However, a role for the direct involvement of the conserved chloride-coordinating Arg500 should not be excluded as it is close to the active site and seen making a water-mediated interaction with Tyr501.

## Discussion

Ac-SDKP is an important substrate of ACE that, through specific inhibition, could lead to beneficial therapeutic effects in the treatment of fibrosis disorders. Therefore, a thorough enzymatic and structural analysis would be useful in assisting our understanding of the requirements for hydrolysis of tetrapeptide substrates. Previous work involving Ac-SDKP kinetics involved the use of paper chromatography or HPLC, neither of which allow for medium to high throughput kinetic characterisation^7^,^38^,^39^. A modified fluorescamine assay was previously applied to ACE substrates of *N*-acetyl-AngI and substance P^40^,^41^, as well as to other enzyme substrates^42^,^43^. Recently, our group employed this derivatising agent to assess simple enzyme activity of a newly identified polymorphism^44^. Here we employed fluorescamine to assess the detailed kinetics of Ac-SDKP processing by ACE.

The N-domain selectivity of Ac-SDKP processing was found to be markedly reduced compared to some previous reports. Literature reports differ on the degree of catalytic efficiencies and selectivities of Ac-SDKP hydrolysis^7^,^38^. The *K*_m_ values determined in this study are generally higher than the original publication presenting Ac-SDKP hydrolysis as a highly N-domain specific process^7^ also determined *K*_m_ values that differ depending on the enzymatic construction and methodology employed^38^. The selectivity of Ac-SDKP reported here for the individual domains (N-domain and C-domain) is only 2-fold. Deddish *et al*. also found a decrease in selectivity, with Ac-SDKP having approximately 8-fold N-selectivity^38^. In this study, the C-domain enzymes had a considerably higher turnover rate than previously described, thus decreasing the overall selectivity. Increased C-domain activity could be due to the assay conditions used here that differs to other studies, where we sought to best mimic the NaCl physiological context (generally 100 mM NaCl). In the original publication of the N-selectivity of Ac-SDKP processing, despite showing that maximal activity of both domains occurs at 100 mM NaCl, a concentration of 50 mM NaCl was used in the assay buffers^7^. Indeed, lower chloride concentrations seemed to result in increased preference for N-domain activity^38^. Thus, a contributor to higher C-domain activity could possibly be due to the use of an optimal chloride concentration for the C-domain^6^,^37^. Interestingly, the *in vivo* N-domain inactivation results in a 4–7 fold increase in plasma Ac-SDKP concentrations^22^,^45^,^46^. This indicates that the selectivity observed in the current report is consistent with more physiologically relevant observations. Importantly, while it seems that the selectivity of Ac-SDKP for the N-domain is less than originally reported, the *in vivo* studies state that the modest increase in serum peptide levels through the use of N- domain selective inhibitors can still possibly provide the desired therapeutic effects.

The structural basis of Ac-SDKP’s preferential hydrolysis by the N-domain was investigated by analysing the molecular interactions of the N-domain with the peptide. The two peptides occupied the same area of the S′ catalytic sub-pocket and displayed noticeable differences in their mode of binding within the S1′ site while showing common features in carboxy-terminal ends binding within with the S2′ sites. This common anchoring in the S2′ site is homologous to that of peptide binding in C-domain ACE^31^, and of the peptide-based N-domain inhibitors RXP 407^33^ and 33RE^35^. Surprisingly, the substrate binding pockets of N-domain ACE do not present any changes at all between the two structures and can accommodate the two very different peptides without any conformational rearrangement (Fig. 1). Interestingly, phosphinic inhibitors were recently showed to fit to the conserved substrate binding pocket of the two domains of ACE and its Drosophila homologue (AnCE) with the enzymes showing little plasticity^47^. This unspecific mechanism of peptide recognition may explain the wide range of substrates cleaved by this enzyme.

Using the structural information above, we were able to generate a model for Ac-SDKP binding into the enzyme active site and revealed the importance of the S2 site in providing possible unique interactions for preferential processing. Further, it suggests a minimal set of amino acids that are responsible for enzyme selectivity that, if properly exploited, could result in domain selective inhibitors and/or drugs. Some of these residues have been implicated in selective inhibitor binding^35^,^36^ and thus this study also serves the prioritisation of optimal interactions with this site. The *N*-acetyl group is easily accommodated in the cavernous S2 site with the primary contacts in this region being carried out by the P2 Ser of the substrate. Our structural model provides visual insight for observations of other kinetic studies, whereby selectivity of Ac-SDKP analogues could be influenced by changing the unprimed substrate amino acids while having benzoyl derivatives (considerably larger than an acetyl group) on the substrate N-terminus^8^,^48^. Taken together, this suggests a limited role of the acetyl group in contributing to preferential processing.

The two active sites within human and bovine somatic ACE exhibit negative co-operativity with certain, but not all, substrates^26^,^27^,^49^,^50^. The current study shows that the catalytic efficiencies of sACE in Ac-SDKP hydrolysis appears overall to be additive between the two domains. Similar to other kinetic observations with physiological substrates, such as Ang(1–9) and BK, the degree of co-operativity between domains in Ac-SDKP processing is considerably less than many synthetic peptides observed previously^27^,^51^. The sACE CC-domain enzyme possessed a catalytic ability per active site similar to the individual domains, indicating an additive effect between the two C-domains. The analysis of the CC-sACE enzyme also indicated that the N-domain has some inhibitory effect on the C-domain in the somatic isoform, an effect noticed in both kinetics of fluorogenic substrates and in the proteolytic shedding of ACE^50^. It seems plausible that such an effect is relevant in the physiological setting as well, possibly either by affecting substrate access, product release, or by reducing the overall dynamics of the domain required for efficient processing.

In summary, the detailed molecular interactions between Ac-SDKP and ACE were characterised. We have found that the tetrapeptide is indeed preferentially processed by the N-domain although this preference is less than some previous reports. The observations of the dipeptide fragments resulting from Ac-SDKP cleavage provided the structural basis to offer a complete model of domain preferential substrate hydrolysis, from peptide recognition by specific domain amino acids to a detailed catalytic mechanism. Further, we emphasize important sets of amino acids: conserved anchoring residues that appear to play a role in ACE substrate binding generally and N-domain unique residues that could provide contacts for preferential positioning. Domain interactions were also shown to influence ACE activity with an observed inhibitory effect of the N-domain over C-domain cleavage of Ac-SDKP. Overall, these findings enhance our understanding of peptide hydrolysis generally and should help the development of N-domain selective inhibitors that could be useful in the treatment of tissue injury and fibrosis.

## Methods

### Enzymes

For single, soluble enzymatic domains: a modified tACE construct, tACEΔ36NJ, that lacks the transmembrane region and unique 36 amino acid N-terminus (and therefore identical to the sACE C-domain; hereon referred to as C-domain) had been generated previously^52^. A soluble form of the N-domain, consisting of amino acids 1 to 629 of somatic ACE (hereon referred to as N-domain), in vector pECE was a kind gift from Dr. Sergei Danilov UIC, Chicago) and was cloned into sequencing vector pBlueScript SK+ (Invitrogen) as previously described^29^,^53^.

The full length domain knock outs of sACE were a kind gift from Dr. Vincent Dive^6^. A full length molecule with only an active N-domain active site (N-sACE) has zinc binding residues His361 and His365 converted to Lys. Similarly a full length molecule with only an active C-domain active site (C-sACE) possesses conversions of the corresponding His residues. The CC-sACE molecule consists of two C-domains joined by the sACE inter-domain linker region and was constructed as described previously^50^. All full length constructs have the complete signal, transmembrane and stalk region corresponding to wild type sACE.

### Expression and purification of enzymes

All enzymes were expressed in Chinese Hamster Ovary (CHO) cells using standard tissue culture approaches as formerly described^54^. All enzymes were purified using lisinopril-Sepharose affinity chromatography as previously described^55^ with the following considerations: single domains were isolated from the harvest medium while full length enzymes were purified from whole cell triton lysates. N-domain constructs required the addition of 800 mM NaCl to medium/lysates for effective purification^56^. ACE activity was detected using the substrate Cbz-Phe-His-Leu (*Z*-FHL)^57^ and pooled enzyme dialysed twice with 2 litres of 5 mM Hepes (pH 7.5). All enzymes were concentrated and stored at 4 °C in 50 mM Hepes (pH 7.5). Enzyme integrity and purity were assessed by SDS-PAGE and subsequent Coomassie staining.

In order to determine loss enzymatic activity due to storage, specific activities were calculated immediately after purification assuming that the enzyme which is eluted off the lisinopril-Sepharose column must be active in order to bind the ligand. Specific activities were re-determined prior to kinetic analysis and active protein concentrations adjusted accordingly.

### Determination of kinetic parameters

A plate adapted fluorescamine assay was employed as published^44^. Thirty microlitres of AcSDKP substrate in Hepes buffer (50 mM Hepes (pH 7.5), 100 mM NaCl, 10 μM ZnSO_4_ buffer), ranging in concentration from 0 to 1000 μM, were warmed to 37 °C in a 96-well plate. The assay was commenced by the addition of 10 μl pre-warmed enzyme (0.2 pmols in Hepes buffer) and incubated for 15 minutes for N-domain and sACE enzymes and 30 minutes for the C-domain enzymes. The assay was stopped by the addition of 50 μl 1 M HCl. The solution was neutralised by the addition of 50 μl 1 M NaOH and the pH increased to 8.3 by the addition of 100 μl 500 mM K_2_HPO_4_/KH_2_PO_4_ buffer pH 8.3. Ten microlitres of fluorescamine (2 mg/ml in acetone, Sigma-Aldrich Co.) was added and the resulting mixture incubated for 3 minutes at room temperature. Fluorescence intensities were measured at λ_ex _= 390 nm and λ_em _= 475 nm using a Cary Eclipse spectrofluorimeter (Varian Inc.). Changes in fluorescence compared to unhydrolysed substrate were converted to reaction velocities by the use of a standard curve obtained by complete AcSDKP hydrolysis and kinetic constants calculated from nonlinear regression analysis (v 4.01, GraphPad Prism^®^). For full length ACE constructs, the presence of 2 moles active sites for every 1 mole of enzyme was taken into account when calculating turnover rates^27^.

### X-ray crystallography

The crystals of N-ACE in complex with the peptides were obtained by co-crystallization with a 2.5 mM concentration of Ac-SDKP (Biorbyt, orb70378). Crystals were grown with 1 μl of the N-ACE:Ac-SDKP sample (protein at 8 mg/ml in 50 mm HEPES, pH 7.5, 0.1 mm PMSF) mixed with an equal volume of reservoir solution consisting of 30% PEG550 MME/PEG20000, 100 mM Tris/Bicine, pH 8.5, and 0.06 M divalent cations (Molecular Dimensions) and suspended above the well as a hanging drop.

X-ray diffraction data were collected on station IO3 at the Diamond Light Source (Oxon, UK) equipped with a PILATUS-6M detector (Dectris, Switzerland). Crystals were kept at constant cryo-temperature (100 K) during data collection. Raw data images were processed and scaled with MOSFLM^58^, and SCALA using the CCP4 suite 6.5^59^. Molecular replacement with the coordinates of N-domain (PDB code 3NXQ)^30^ was used to determine initial phases for structure solution in PHASER^60^. The working models were refined using REFMAC5^61^ and manually adjusted with COOT^62^. Water molecules were added at positions where *F*_*o*_ − *F*_*c*_ electron density peaks exceeded 3σ, and potential hydrogen bonds could be made. Validation was performed with MOLPROBITY^63^. Crystallographic data statistics are summarized in Table 1. All figures were drawn with PyMOL (Schrödinger, LLC, New York). Hydrogen bonds were verified with the program LigPlot^+^^64^.

## Additional Information

**How to cite this article**: Masuyer, G. *et al*. Structural basis of Ac-SDKP hydrolysis by Angiotensin-I converting enzyme. *Sci. Rep*. **5**, 13742; doi: 10.1038/srep13742 (2015).

## Figures and Tables

**Table 1:** Crystallographic statistics of N-domain ACE in complex with Ac-SDKP fragments.

	N-dom:Ac-SD	N-dom:KP
Resolution (Å)	1.8	1.8
Space group	*P1*	
Cell dimensions and angles Number of molecules/asymmetric unit	a = 73, b = 77, c = 83 Å; α = 89, β = 64, γ = 75° 2	a = 73, b =102, c = 115 Å; α = 85, β = 86, γ = 82° 4
Total/Unique reflections	419,370 130,327	936,596 293,868
Completeness (%)^a^	90 (58)	97 (96)
*R*_merge_^a^^,^^b^	10.9 (64.9)	10.3 (70.2)
*R*_pim_^a^^,^^c^	7.1 (43.9)	6.8 (45.7)
I/σ(I)^a^	6.1 (1.6)	6.2 (1.5)
*R*_*cryst*_^d^	20.2	20.7
*R*_free_^e^	22.8	23.5
RMSD in bond lengths (Å)	0.010	0.007
RMSD in bond angles (°)	1.37	1.18
B- factor statistics (Å^2^)		
Protein all atoms (per chain)	24.7/30.2	30.3/31.3/25.9/25.8
Protein main chain atoms (per chain)	23.8/29.4	29.6/30.7/25.2/25.2
Protein side chain atoms (per chain)	25.7/31.0	31.0/31.8/26.5/26.5
Peptide atoms (per chain)	42.0/39.9	25.9/26.0/25.6/24.1
Solvent atoms	32.2	32.1
Zn^2+^/Cl^−^ ions	17.8/19.1	20.6/20.8
Glycosylated carbohydrate atoms	64.3	56.1
Ramachandran statistics (Molprobity)		
Favored	98.1%	98.1%
Outliers	0.3%	0.1%
PDB code	**4ufa**	**4ufb**

^a^Values in parentheses refer to the highest resolution shell.

^b^*R*_merge _= ΣΣ_*i*_|*I*_*h*_ − *I*_*hi*_|/ΣΣ_*i*_*I*_*h*_, where *I*_*h*_ is the mean intensity for reflection *h*.

^c^*R*_pim _= Σ_*h*_ (1/*n*_*h*_ − 1) Σ_*l*_ |*I*_*hl*_ − (*I*_*h*_)|/Σ_*h*_Σ_*l*_(*I*_*h*_).

^d^*R*_cryst _= Σ‖*F*_*o*_| − |*F*_*c*_‖/Σ|*F*_*o*_|, where *F*_*o*_ and *F*_*c*_ are measured and calculated structure factors, respectively.

^e^*R*_free _= Σ‖*F*_*o*_| − |*F*_*c*_|/Σ|*F*_*o*_|, calculated from 5% of the reflections selected randomly and omitted during refinement.

**Table 2:** Kinetic parameters of Ac-SDKP hydrolysis by different ACE enzymes.

Enzyme	*k*_cat_ (s^−1^)	*K*_m_ (μM)	*k*_cat_/*K*_m_ (s^−1^.M^−1^)
N-dom	14.2 ± 1.2	199.6 ± 25.5	(0.72 ± 0.06) × 10^5^
C-dom	4.8 ± 0.6	138.2 ± 20.3	(0.35 ± 0.01) × 10^5^
sACE	12.7 ± 0.4	239.5 ± 29.4	(0.54 ± 0.06) × 10^5^
N-sACE	34.0 ± 3.9	493.8 ± 79.3	(0.70 ± 0.04) × 10^5^
C-sACE	8.2 ± 0.5	436.3 ± 33.4	(0.188 ± 0.002) × 10^5^
CC-sACE	19.4 ± 0.8	503.8 ± 31.6	(0.39 ± 0.03) × 10^5^

Turnover rates were normalised to activity per unit active site for enzymes containing two functional domains (sACE and CC-sACE).

**Table 3:** Alignment of ACE residues involved in direct interactions with Ac-SDKP compared to the C-domain ACE-AngII and N-domain ACE-RXP407 complexes.

Position	N-AcSDKP		Ang II^31^		RXP407 inhibitor^30^	
	N-domainACE	Correspondingresidues in C-ACE	Correspondingresidues in N-ACE	C-domainACE	N-domainACE	Correspondingresidues in C-ACE
S2			D336	D358		
S2	Y369	F391			Y369	F391
S2	H388	H410				
S1	A334	A356	A334	A356	A334	A356
S1	E362	E384	E362	E384	E362	E384
S1	T496	V518				
S1′	H331	H353	H331	H353	H331	H353
S1′	A332	A354	A332	A354	A332	A354
S1′	T358	V518				
S1′	H491	H513	H491	H513	H491	H513
S1′	Y501	Y523	Y501	Y523	Y501	Y523
S2′	Q259	Q281	Q259	Q281	Q259	Q281
S2′	K489	K511	K489	K511	K489	K511
S2′	Y498	Y520	Y498	Y520	Y498	Y520

Structural comparison of the two domains of ACE in complex with their preferred substrate (Ac-SDKP for N-domain ACE, and Ang II for C-domain ACE) highlights the common mechanism of peptide recognition. The model of N-domain ACE with its full Ac-SDKP substrate indicates that further direct interactions with N-ACE specific residues are the basis for its domain partiality. Additionally, several potential interactions were distinguished that were not observed in the N-ACE complex structure with RXP407, the most selective N-ACE inhibitor to date.
